# Copy Number Variants in German Patients with Schizophrenia

**DOI:** 10.1371/journal.pone.0064035

**Published:** 2013-07-02

**Authors:** Lutz Priebe, Franziska Degenhardt, Jana Strohmaier, René Breuer, Stefan Herms, Stephanie H. Witt, Per Hoffmann, Rebecca Kulbida, Manuel Mattheisen, Susanne Moebus, Andreas Meyer-Lindenberg, Henrik Walter, Rainald Mössner, Igor Nenadic, Heinrich Sauer, Dan Rujescu, Wolfgang Maier, Marcella Rietschel, Markus M. Nöthen, Sven Cichon

**Affiliations:** 1 Institute of Human Genetics, University of Bonn, Bonn, Germany; 2 Department of Genomics, Life and Brain Center, University of Bonn, Bonn, Germany; 3 Department of Genetic Epidemiology in Psychiatry, Central Institute of Mental Health, Medical Faculty Mannheim/Heidelberg University, Mannheim, Germany; 4 Institute for Genomic Mathematics, University of Bonn, Bonn, Germany; 5 Channing Division of Network Medicine, Brigham and Women's Hospital and Harvard Medical School, Boston, Massachusetts, United States of America; 6 Institute of Medical Informatics, Biometry, and Epidemiology, University Duisburg-Essen, Essen, Germany; 7 Department of Psychiatry and Psychotherapy, Central Institute of Mental Health, Medical Faculty Mannheim/Heidelberg University, Mannheim, Germany; 8 Department of Psychiatry and Psychotherapy, Charité Campus Mitte, Berlin, Germany; 9 Department of Psychiatry, University of Bonn, Bonn, Germany; 10 Department of Psychiatry and Psychotherapy, Jena University Hospital, Jena, Germany; 11 Molecular and Clinical Neurobiology, Department of Psychiatry, Ludwig-Maximilians-University, Munich, Germany; 12 Department of Psychiatry, University of Halle-Wittenberg, Halle, Germany; 13 German Center for Neurodegenerative Diseases (DZNE), Bonn, Germany; 14 Institute of Neuroscience and Medicine (INM-1), Structural and Functional Organisation of the Brain, Genomic Imaging, Research Centre Juelich, Juelich, Germany; 15 Division of Medical Genetics, University Hospital and Department of Biomedicine, University of Basel, Basel, Switzerland; University of Wuerzburg, Germany

## Abstract

Large rare copy number variants (CNVs) have been recognized as significant genetic risk factors for the development of schizophrenia (SCZ). However, due to their low frequency (1∶150 to 1∶1000) among patients, large sample sizes are needed to detect an association between specific CNVs and SCZ. So far, the majority of genome-wide CNV analyses have focused on reporting only CNVs that reached a significant P-value within the study cohort and merely confirmed the frequency of already-established risk-carrying CNVs. As a result, CNVs with a very low frequency that might be relevant for SCZ susceptibility are lost for secondary analyses. In this study, we provide a concise collection of high-quality CNVs in a large German sample consisting of 1,637 patients with SCZ or schizoaffective disorder and 1,627 controls. All individuals were genotyped on Illumina's BeadChips and putative CNVs were identified using QuantiSNP and PennCNV. Only those CNVs that were detected by both programs and spanned ≥30 consecutive SNPs were included in the data collection and downstream analyses (2,366 CNVs, 0.73 CNVs per individual). The genome-wide analysis did not reveal a specific association between a previously unknown CNV and SCZ. However, the group of CNVs previously reported to be associated with SCZ was more frequent in our patients than in the controls. The publication of our dataset will serve as a unique, easily accessible, high-quality CNV data collection for other research groups. The dataset could be useful for the identification of new disease-relevant CNVs that are currently overlooked due to their very low frequency and lack of power for their detection in individual studies.

## Introduction

Schizophrenia (SCZ) is a severe and debilitating neuropsychiatric disorder with a lifetime prevalence of 0.5–1%. Based on twin studies, it was estimated that SCZ has a heritability of ∼80% [1]. Several large copy number variants (CNVs) have been identified as risk factors for SCZ susceptibility [2]–[14]. Among patients with SCZ, the frequency of such CNVs is low and ranges between 1∶150 and 1∶1,000 [15]. The majority of published CNV studies have focused on microduplications and microdeletions that reach a nominally significant P-value, and thus these studies only report the frequency of already-established risk-bearing CNVs. Therefore, very rare CNVs that might be disease-relevant go undetected and unreported due to the limited sample sizes of current studies and are consequently lost for follow-up studies. To help change this, we set out to compile a concise collection of high-quality CNV calls in a clinically well-characterized sample of 1,637 patients with SCZ or schizoaffective disorder (SCZA). Additionally, using data from 1,627 controls we (i) performed a genome-wide CNV analysis, and (ii) checked the frequency of CNVs previously reported to be associated with schizophrenia.

## Materials and Methods

### Ethics statement

Each participant provided written informed consent prior to inclusion and all aspects of the study complied with the Declaration of Helsinki. The study was approved by the ethics committees of all study centers: Ethics Committee of the Rheinische Friedrich-Wilhelms-University Medical School in Bonn, Ethics Committee “Medizinische Ethik-Kommission II” University of Heidelberg, Ethics Committee of the Friedrich-Schiller-University Medical School in Jena, and Ethics Committee of the Ludwig-Maximilians-University Munich.

In our study, we only included patients who had full capacity to consent. The ability to consent was determined by the referring psychiatrist. We did not enclose patients who were under care or had a legal guardian. In exceptional cases, a patient with a legal guardian wished to participate and we did not want to discriminate against him/her by denying participation to the study. In these rare cases, written informed consent was obtained by both the patient and the legal guardian. At the recruiting site in Jena, patients with a legal guardian, who wanted to be included in the study, had to have full capacity to consent and no legal obligation to obtain additional permission by their legal guardian. All potential participants who declined to participate (for any reason) were eligible for treatment and were not disadvantaged in any other way by not participating in the study.

### Sample description

All individuals were of German descent according to self-reported ancestry. A total of 1,831 patients were recruited from consecutive admissions to psychiatric inpatient units. A lifetime “best estimate” diagnosis [16] of SCZ or SCZA according to DSM-IV criteria [17] was assigned on the basis of the Structured Clinical Interview [18] or the OPCRIT [19], medical records, and family history. A total of 1,643 controls were included. These are described in detail elsewhere [20].

### Genotyping and quality control

Venous blood samples were obtained from all participants. These were genotyped separately using the following Illumina BeadChips: HumanHap550v3, Human610-Quadv1, and Human660W-Quad. Only those markers common to all three chips were analyzed. In total we analyzed 546,137 markers. To avoid technical artifacts in CNV calling, stringent quality control criteria were applied prior to computational CNV prediction, as described in Degenhardt et al. [20].

### CNV detection and CNV quality control criteria

Detailed information on CNV detection and CNV quality control is provided elsewhere [20]. In brief, to identify potential CNVs, the BeadChip data of each participant was analyzed with QuantiSNP (version 2.1, http://www.well.ox.ac.uk/QuantiSNP; [21]) and PennCNV (version 2010May01, http://www.openbioinformatics.org/penncnv/; [22]). Individuals were excluded if their standard deviation from the log R ratio calculated over all SNPs exceeded 0.30. CNVs were required to have a minimum of 30 consecutive SNPs and a log Bayes Factor (lBF; QuantiSNP) or confidence value (PennCNV) of at least 30.

### Merging PennCNV and QuantiSNP CNV data

Only those CNVs that passed our filter criteria and that were called by both QuantiSNP and PennCNV were included in our analysis. All CNVs that are listed in the supplement were visually inspected and confirmed using Illumina's GenomeStudio Genotyping Module (version 1.6.3, http://www.illumina.com/pages.ilmn?ID=169; table S1). In cases where the two programs identified differing CNV breakpoints, only the overlapping CNV was reported. Rarely, one program detected one large CNV whereas the other program identified two smaller CNVs. Based on the visual inspection of the CNV and the SNP coverage in the affected CNV region, the predicted CNV that was more likely to be genuine was selected.

All CNVs that passed the stringent filter criteria were presumed to be genuine CNVs. In two previous studies, 100% of our putative CNVs were shown to be technically verifiable when our filter criteria were employed [20], [23].

### Statistical analysis of CNVs

The datasets generated by QuantiSNP and PennCNV were analyzed using PLINK (version 1.07, http://pngu.mgh.harvard.edu/~purcell/plink/index.shtml [24]). To test for associations between SCZ and CNVs in specific chromosomal regions, Fisher's exact test was applied to calculate P-values and odds ratios.

### CNVs in regions previously associated with SCZ

The presence in our dataset of CNVs previously shown to be associated with SCZ in at least two independent studies was examined. To be included in our analysis, the CNVs derived from our dataset had to overlap ≥80% with the reported CNVs. CNVs in 16p13.11 had to overlap ≥80% with interval II, as described by Ingason et al. [10]. For CNVs in 2p16.3, we considered all CNVs that affected the gene neurexin-1 and spanned ≥10 consecutive SNPs. We allowed for the inclusion of smaller deletions and duplications because smaller CNVs in *NRXN1* were previously reported to be associated with schizophrenia [5]. All CNVs affected *NRXN1* directly and only five out of 11 patients had a CNV smaller than 30 SNPs. Each CNV was visually confirmed in the GenomeStudio.

## Results

### Data collection of high-quality CNVs

After application of all filter criteria, data from 1,637 patients and 1,627 controls remained. Using QuantiSNP, 2,487 CNVs passed our set of filter criteria (0.76 CNVs per individual); for PennCNV, 2,430 CNVs passed all filters (0.74 CNVs per individual). After merging the two datasets and removing those CNVs that were detected by only one program, 2,366 CNVs (0.73 CNVs per individual) remained in our dataset. All CNVs derived from our patients are provided in table S1. Individual genotype and intensity data can be obtained on a collaborative basis.

### Genome-wide association analysis

We performed a genome-wide analysis to uncover associations between specific CNVs and SCZ, but we did not detect any specific CNVs to be nominally significantly associated with the disorder. However, it is noteworthy that, in our dataset, a CNV had to be identified in at least six patients and in no control to reach a nominally significant P-value.

### CNVs in regions previously associated with SCZ

In total, we analyzed 11 CNV regions (CNVRs) that are established risk factors for SCZ susceptibility and detected a higher proportion of CNVs overlapping ≥80% with those regions in patients than in the controls. We identified 20 microdeletions and five microduplications in 1,637 patients as compared with 12 microdeletions in 1,627 controls (see Table 1, P-value: 0.051). No duplication in any of the 11 CNVRs was detected among the controls. In our dataset, none of the specific CNVs was individually significantly associated with SCZ. None of our patients carried a CNV in 3q29, 15q13.3, 17p12, or 17q12. However, in 1q21.1, 2p16.3, and 22q11.2, we identified a higher proportion of microdeletions in patients as compared with controls (3/1,637 in patients versus 1/1,627 in controls in 1q21.1; 10/1,637 in patients versus 5/1,627 in controls in 2p16.3, and 2/1,637 in patients versus 0/1,627 in controls in 22q11.2). We detected a microduplication in 7q36.3 and 16p11.2 in the patients, whereas neither of these microduplications was detected in the controls. In 15q11.2 we identified a higher proportion of microdeletions in controls as compared with patients (2/1,637 in patients versus 3/1,627 in controls. Phenotypic information for the CNV carriers is provided in Table 2.

**Table 1:** CNVs in regions previously associated with SCZ.

Locus	Position	Reference	Patients (n = 1,637)	Controls (n = 1,627)	P	OR	P-del	OR-del	P-dup	OR-dup
1q21.1	144.94–146.29	[2], [3], [7]	3 del	1 del	**0.62**	2.98	**0.62**	2.98	–	–
2p16.3 (*NRXN1*, gene affecting)	49.90–51.50	[4], [5], [7]	10 del, 1 dup	5 del, 0 dup	**0.21**	2.19	**0.30**	1.99	**1**	>1
3q29	197.2–198.83	[6], [7], [9]	–	–	–	–	–	–	–	–
7q36.3	158.51–158.63	[7], [9]	1 dup	–	**1**	>1	–	–	**1**	>1
15q11.2	20.31–20.78	[3], [11], [12]	2 del	3 del	**0.69**	0.66	**0.69**	0.66	–	–
15q13.3	28.72–30.30	[2], [3], [7], [9], [12]	–	–	**–**	–	**–**	–	–	–
16p13.11 (affecting interval II)	15.38–16.19	[10], [11], [12]	3 del, 2 dup	3 del, 0 dup	**0.73**	1.66	**1**	0.99	**0.5**	>1.99
16p11.2	29.57–30.21	[7], [8], [9]	1 dup	–	**1**	>1	–	–	**1**	>1
17p12	14.00–15.40	[11], [12]	–	–	–	–	–	–	–	–
17q12	31.89–33.28	[12], [13]	–	–	–	–	–	–	–	–
22q11.21	17.00–19.80	[3], [7], [9], [14]	2 del	–	**0.50**	>1.99	**0.50**	>1.99	–	–
sum	–	–	20 del, 5 dup	12 del, 0 dup	**0.05**	2.09	**0.21**	1.66	**0.06**	>4.98

SCZ, schizophrenia; del, microdeletion; dup, microduplication; P, P-value; OR, odds ratio. Position according to NCBI built 36; all P-values and ORs were calculated using Fisher's exact test.

Two of the patients with a deletion in 1q21.1 and one patient with a deletion in 15q11.2 were already reported by Stefansson et al. [3], three patients with a deletion in Neurexin were included in Rujescu et al. [5] and two patients with a deletion and one patient with a duplication in 16p13.11 were part of the study performed by Ingason et al. [10].

**Table 2:** Phenotypic information of patients with a CNV in regions previously associated with SCZ.

Locus	Patient	Chr	Start	Stop	CNV	Diagnosis	Gender	AAO (years)	Familiy history SCZ	Education
**1q21**	671	1	144,106,312	147,427,061	DEL	295.3	f	33	no	passed exams after nine years of schooling
	17	1	144,967,972	147,331,145	DEL	295.3	m	20	no	.
	579	1	144,967,972	146,293,282	DEL	295.3	f	16	no	passed exams after nine years of schooling
**2p16.3**	68	2	50,658,484	50,847,759	DEL	295.7	m	14	no	passed exams after nine years of schooling
	382	2	50,797,032	50,895,846	DEL	295.4	f	32	.	passed A levels (13 years of schooling)
	702	2	50,836,690	50,936,258	DEL	295.3	f	20	no	high-school diploma (10 years of schooling)
	720	2	50,850,456	51,225,851	DEL	295.3	f	27	yes	passed exams after nine years of schooling
	502	2	50,870,615	50,981,293	DEL	295.7	m	45	no	passed A levels (13 years of schooling)
	288	2	51,068,497	51,255,180	DEL	295.3	m	22	no	passed exams after nine years of schooling
	779	2	50,716,777	50,756,435	DEL	295.3	m	18	no	passed A levels (13 years of schooling)
	812	2	50,735,657	50,800,548	DEL	295.3	f	34	no	passed A levels (13 years of schooling)
	813	2	50,735,657	50,800,548	DEL	295.3	m	26	no	passed A levels (13 years of schooling)
	273	2	50,750,960	50,829,989	DUP	295.3	m	30	yes	passed A levels (13 years of schooling)
	361	2	50,912,249	50,948,557	DEL	295.3	m	44	no	left school after nine years, sat no examinations
**7q36**	228	7	158,456,556	158,812,247	DUP	295.1	f	22	.	left school after nine years, sat no examinations
**15q11.2**	383	15	20,306,549	20,685,685	DEL	295.4	f	32	no	passed exams after nine years of schooling
	107	15	20,317,047	20,778,963	DEL	295.x	m	18	no	.
**16p13.11**	149	16	15,032,942	15,988,624	DEL	295.x	f	30	.	.
	558	16	15,032,942	16,197,033	DEL	295.6	f	23	no	high-school diploma (10 years of schooling)
	332	16	15,036,960	16,197,033	DUP	295.3	m	30	no	.
	668	16	15,387,380	16,197,033	DEL	295.6	m	18	no	passed exams after nine years of schooling
	555	16	15,387,380	18,174,650	DUP	295.1	m	17	no	high-school diploma (10 years of schooling)
**16p11.2**	745	16	29,563,365	30,085,308	DUP	295.6	f	19	yes	school for children with special needs, sat no examinations
**22q11.2**	252	22	17,257,787	19,792,353	DEL	295.3	f	.	.	passed exams after nine years of schooling
	775	22	17,257,787	19,792,353	DEL	295.3	f	16	yes	passed exams after nine years of schooling

Chr, chromosome; DEL, deletion; DUP, duplication; Diagnosis, DSM-IV diagnosis; f, female; m, male; AAO, age-at-onset; Family history SCZ, any fist degree relative with schizophrenia; ., missing data; Position according to NCBI built 36.

## Discussion

In this study, we provide a unique, high-quality CNV data collection in a clinically well characterized sample of 1,637 patients with SCZ and SCZA. Applying conservative, stringent filter criteria is expected to reduce type I errors, whereas type II errors are expected to increase. This strategy benefitted the genome-wide CNV dataset for two main reasons: (i) larger CNVs were selected for and these were more likely to have a stronger impact on the phenotype than smaller CNVs, and (ii) only CNVs that spanned a fairly large number of consecutive SNPs and that were detected by two CNV programs were selected, which increased the likelihood of obtaining genuine CNVs. In our previous experience, all microduplications and microdeletions fulfilling our quality criteria can be successfully verified by quantitative real-time PCR using TaqMan Copy Number Assays (Applied Biosystem, Foster City, CA, USA) or Fast SYBR® Green (Life Technologies, Carlsbad, California, USA), or pre-designed SALSA® MLPA® (Multiplex Ligation-dependent Probe Amplification) kits (MRC Holland, Amsterdam, the Netherlands) [20], [23]. This high CNV reliability is important as we hope the dataset will serve as a resource for other investigators who might wish to combine their own data with our data with the aim of achieving a higher detection rate for associations between individual CNVs and this disease.

We checked our dataset for the presence of CNVs in regions previously associated with SCZ. Even though none of the CNVs in the 11 analyzed CNVRs reached a significant P-value, we found a higher frequency of CNVs in 1q21.1, 2p16.3, 7q36.3, 16p13.11, 16p11.2, and 22q11.2 in patients than in the controls. Therefore, our study provides additional support that CNVs in these regions are genetic risk factors for SCZ. None of our patients carried a CNV in 3q29, 15q13.3, 17p12, or 17q12. The frequency of SCZ-associated CNVs is estimated to be low (e.g. <1% for CNVs in 3q29 and 17q12; [11], [13]). Therefore, one likely explanation for a lack of CNVs in these regions is that our dataset, despite its reasonable size, might still not be sufficiently powered to detect very rare CNVs. However, our study confirms the low frequency of risk-carrying CNVs in controls.

The main limitation of the present study is, at the same time, its greatest strength. Applying stringent filter criteria could have led to the removal of smaller, and potentially true, CNVs from our dataset. Conversely, this approach ensured that the reported CNVs were of high quality.

## Supporting Information

Table S1 **List of CNVs in patients.**
(XLSX)
